# Placental Glucose Transfer: A Human In Vivo Study

**DOI:** 10.1371/journal.pone.0117084

**Published:** 2015-02-13

**Authors:** Ane M. Holme, Marie Cecilie P. Roland, Bjørg Lorentzen, Trond M. Michelsen, Tore Henriksen

**Affiliations:** 1 Department of Obstetrics, Oslo University Hospital, Oslo, Norway; 2 Norwegian National Advisory Unit on Women’s Health, Oslo University Hospital, Oslo, Norway; 3 University of Oslo, Oslo, Norway; University of Oxford, UNITED KINGDOM

## Abstract

**Objectives:**

The placental transfer of nutrients is influenced by maternal metabolic state, placenta function and fetal demands. Human in vivo studies of this interplay are scarce and challenging. We aimed to establish a method to study placental nutrient transfer in humans. Focusing on glucose, we tested a hypothesis that maternal glucose concentrations and uteroplacental arterio-venous difference (reflecting maternal supply) determines the fetal venous-arterial glucose difference (reflecting fetal consumption).

**Methods:**

Cross-sectional in vivo study of 40 healthy women with uncomplicated term pregnancies undergoing planned caesarean section. Glucose and insulin were measured in plasma from maternal and fetal sides of the placenta, at the incoming (radial artery and umbilical vein) and outgoing vessels (uterine vein and umbilical artery).

**Results:**

There were significant mean (SD) uteroplacental arterio-venous 0.29 (0.23) mmol/L and fetal venous-arterial 0.38 (0.31) mmol/L glucose differences. The transplacental maternal-fetal glucose gradient was 1.22 (0.42) mmol/L. The maternal arterial glucose concentration was correlated to the fetal venous glucose concentration (r = 0.86, *p*<0.001), but not to the fetal venous-arterial glucose difference. The uteroplacental arterio-venous glucose difference was neither correlated to the level of glucose in the umbilical vein, nor fetal venous-arterial glucose difference. The maternal-fetal gradient was correlated to fetal venous-arterial glucose difference (r = 0.8, *p*<0.001) and the glucose concentration in the umbilical artery (r = −0.45, *p* = 0.004). Glucose and insulin concentrations were correlated in the mother (r = 0.52, *p* = 0.001), but not significantly in the fetus. We found no significant correlation between maternal and fetal insulin values.

**Conclusions:**

We did not find a relation between indicators of maternal glucose supply and the fetal venous-arterial glucose difference. Our findings indicate that the maternal-fetal glucose gradient is significantly influenced by the fetal venous-arterial difference and not merely dependent on maternal glucose concentration or the arterio-venous difference on the maternal side of the placenta.

## Introduction

Fetal development comprises the entire intrauterine process of differentiation, growth and maturation between conception and birth. The conditions under which a fetus develops are recognised to have major impact on the future health of the newborn child [1]. The developmental environment of the fetus is dependent on maternal nutritional and metabolic state [2, 3] and on the placental function as most nutrients must be transported from the maternal circulation across the placenta to the fetus. The placenta also secretes hormones and growth factors that change maternal metabolism profoundly [4]. Therefore, placenta governs to a large extent the environment in which the fetus develops and grows.

Increasing maternal fasting glucose levels are associated with higher birthweight, neonatal fat percentage and risk of large babies. [2, 3, 5–7]. Glucose is the main source of energy for the fetus and the placenta. Under normal conditions the fetus has no significant gluconeogenesis and is dependent on glucose transfer from the mother [8]. Glucose is transferred over the placenta by facilitated diffusion, via glucose transport proteins belonging to the GLUT-family [9]. Human studies have demonstrated a robust correlation between the maternal and fetal glucose levels [10]. The Pedersen hypothesis states that high maternal glucose levels lead to high fetal glucose and insulin levels [11]. The current clinical understanding is that the consequence is increased glucose consumption and fetal growth. However experimental data indicate that fetal glucose consumption is affected by other factors than maternal glucose supply alone. The net transfer of glucose over the placenta is thought to be influenced by the maternal-fetal glucose concentration gradient, surface area, transporter density and glucose metabolism of the placenta as well as blood flow [9, 12–15]. The maternal-fetal gradient can be increased by higher level of glucose on the maternal side which is clinically what we monitor and if necessary regulate. The maternal-fetal gradient will however also increase with lower glucose levels on the fetal side of the placenta. [12, 13]. Fundamental studies on pregnant ewes and in vitro perfusion studies indicate that fetal glucose uptake is influenced by fetal glucose levels as well as maternal glucose levels [12, 13, 16]. However, extrapolation to human in vivo condition is hampered by the considerable anatomical and functional differences in the fetoplacental unit among mammalian species. Interestingly, studies of GLUT transporter densities showed different responses to hyperglycaemia depending on whether the exposure was in vivo or in vitro [15]. This observation probably reflects that in vivo there are multiple factors influencing transporter expression and activity. Human in vivo studies of placental physiology are scarce as the placenta is largely unavailable during pregnancy. Glucose and insulin concentrations in maternal and fetal circulations are reported in smaller human studies, of which several were performed during the stress of vaginal delivery [17–20].

Increased insight into the interplay between maternal supply, placental transfer and fetal demand and consumption of nutrients in humans requires a model that provides data from all these three compartment simultaneously. We believe part of this information can be gained by measuring levels of nutrients in the arteries and veins on both the maternal and fetal side of the placenta simultaneously. Calculating the arterio-venous differences and the transplacental gradient gives an opportunity to study how placental uptake and transfer of nutrients are altered by maternal and fetal factors. Thus, our first aim was to establish the 4-vessel procedure for collection of these arterial and venous blood samples from both sides of the placenta. The second aim was to describe placental glucose transfer per litre blood passing by determining the uteroplacental arterio-venous (a-v) difference (reflecting the uteroplacental uptake), the fetal venous-arterial (v-a) difference (reflecting the fetal consumption) and the maternal-fetal gradient (reflecting the transplacental transfer). We tested the hypothesis that the fetal v-a glucose difference is mainly determined by maternal glucose concentration and the uteroplacental a-v glucose difference.

## Materials and Methods

### Ethics Statement

All participants signed a written informed consent. The study was approved by the relevant board at our institution and the Regional Committee for Medical and Health Research Ethics, Southern Norway 2419/2011.

### Design and study population

A cross-sectional in vivo study consisting of 40 women with uncomplicated pregnancies undergoing planned caesarean section. Healthy, non-smoking women with singleton pregnancy were included at Oslo University Hospital from October 2012 until April 2013.

Exclusion criteria were pre-existing morbidity, medication and pregnancy complications. Gestational diabetes not requiring insulin treatment was not an exclusion criterion, based on the rationale that there are no clear metabolic distinction between this group and those defined not to have diabetes. Contractions prior to scheduled caesarean section lead to exclusion.

### Data collection

Clinical data was collected at inclusion. The women were weighed on an impedance scale (Tanita Body composition Analyser, Tokyo, Japan) at the day of delivery. First trimester BMI was calculated based on the first recorded weight, of which 14 were self reported. Gestational weight gain was calculated as the difference between weight at delivery and first trimester.

Caesarean section was performed in spinal anaesthesia (bupivacaine10 mg, fentanyl 20 μg). The women fasted for at least 8 hours and did not receive intravenous glucose infusions. When the uterine vein was exposed immediately before uterine incision blood samples were obtained from a uterine vein on the anterolateral surface of the uterus, intentionally on the same side as placenta. Simultaneously, blood was drawn form the radial artery and from a venous catheter in the medial cubital vein or dorsal vein network of the hand. Blood samples were obtained from the umbilical artery and vein immediately after cord clamping, but before delivery of the placenta.

We used Safety Blood Collection set with Luer Adapter coupled on sterile syringes. Blood samples were immediately transferred to ethylenediaminetetraacetic acid vacutainers (Greiner bio-one, Kremsmunster, Austria), and kept on ice. They were centrifuged at 6°C, 2500 G for 20 minutes, before the supernatants were carefully removed, leaving 0,5 ml to assure platelet free plasma, and stored at- 80°C.

Assays were performed by accredited laboratories according to standard laboratory methods (Department of Medical Biochemistry, Oslo University Hospital, Rikshospitalet) during three following days. Glucose was measured by the hexokinase/glucose-6-phosphate dehydrogenase enzymatic in vitro test (Roche, Mannheim, Germany). Insulin was analysed using the electro-chemiluminescence immunoassay (Roche, Mannheim, Germany).

We assumed similar blood composition in the radial and the uterine artery. Thus, the uteroplacental a-v difference was calculated as the difference in glucose concentration between the radial artery and the uterine vein. The fetal v-a glucose difference was calculated as the difference in glucose concentration between the umbilical vein (transporting blood from the placenta to the fetus) and the umbilical artery (transporting blood from the fetus to the placenta). The maternal-fetal glucose gradient was calculated as the difference in glucose between the maternal radial artery and the umbilical artery.

### Statistics

Descriptive data were reported as mean values with standard deviations (SD) or numbers with percentages as appropriate. Glucose values were reported as mean values (SD). Comparisons between concentrations of glucose in maternal and fetal vessels, respectively, were performed by paired t-tests. Due to skewed distributions, insulin concentrations were reported as median values with inter quartile ranges (IQR) and comparisons of related samples were performed by Wilcoxon sign rank test. Correlations were reported as Pearson’s correlation coefficient if one variable was normally distributed, whereas Spearman’s rank correlation coefficient was used in the case of correlation between two skewed variables. A two-sided *p*-value < 0.05 was considered significant. All analyses were performed using Statistical Package for the Social Sciences, Version 18.0 (SPSS Inc., Hong Kong).

## Results

**Fig 1:**
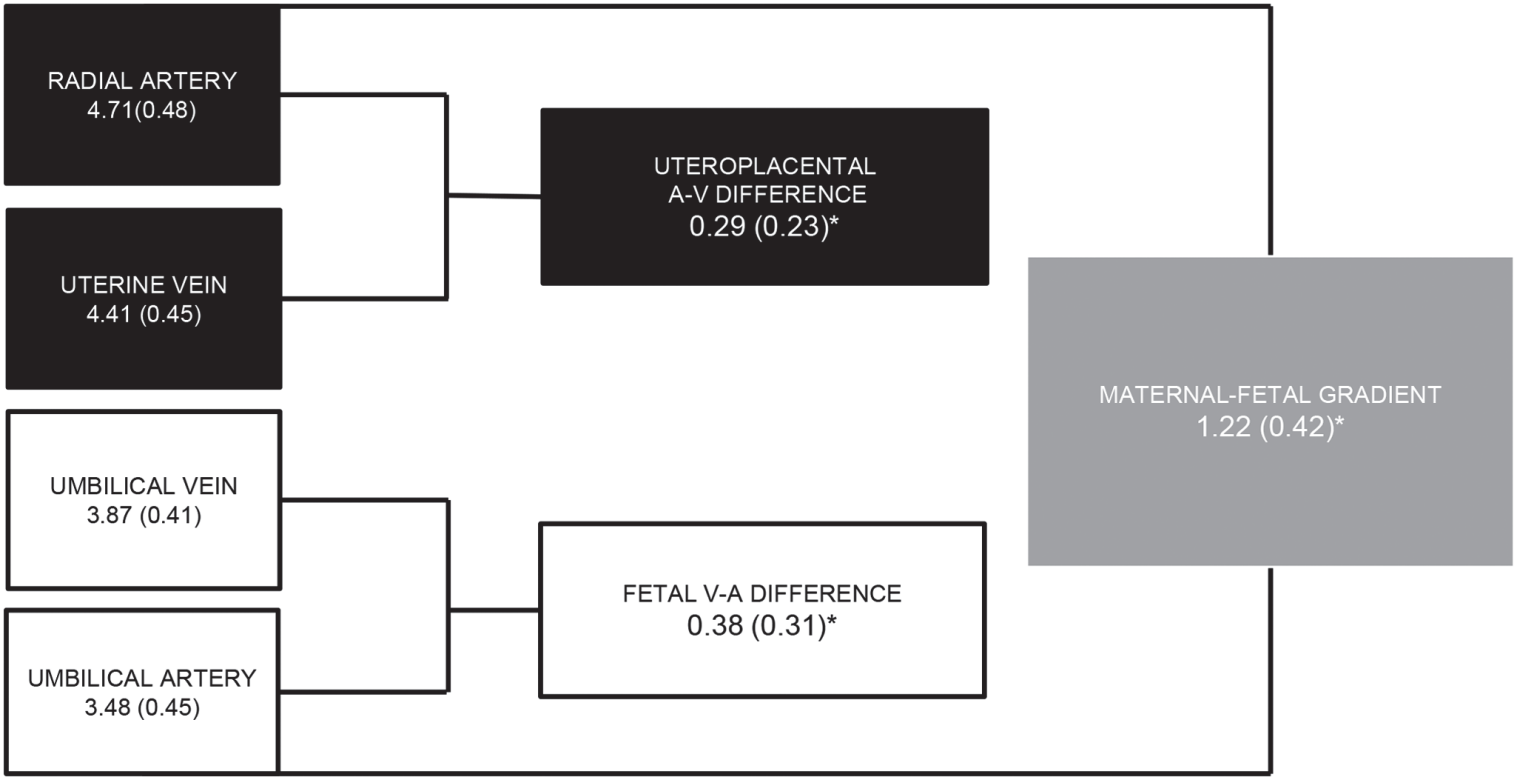
Glucose concentrations and gradients (mmol/L). Glucose concentrations were measured in the arteries and veins on the maternal and fetal side of the placenta. *p<0.001, paired t-test. Calculated parameters and their interpretations: Uteroplacental a-v difference = [Radial artery]-[Uterine vein] Reflects uteroplacental uptake per litre blood passing. Fetal v-a difference = [Umbilical vein]-[Umbilical artery]. Reflects fetal consumption per litre blood passing. Maternal-fetal gradient = [Radial artery]-[Umbilical artery]. Reflects transplacental transfer per litre blood passing.

Characteristics of the participants are shown in Table 1. The glucose concentrations including the concentration differences derived from them and their interpretation are shown in Fig. 1.

**Table 1:** Maternal and neonatal characteristics.

	**n**	**%**	**Mean**	**(SD)**	**Range**
**Mothers**	40				
Age (years)			36.3	(3.2)	30–43
Para 0	10	25			
Para 1	20	50			
Para>1	10	25			
BMI first trimester			24.0	(3.7)	17.6–38.6
BMI at delivery			29.2	(4.2)	22.4–41.2
Weight gain (kg)			14.1	(4.0)	7.3–23.8
Married or partnership	40	100			
Higher education (≥15 years)	36	92.3			
Smoking during pregnancy^a^	0	0			
Gestational diabetes^b^	1	2.5			
In vitro fertilization	4	10			
**Infants**	40				
Gestational age (weeks)			39.4	(0.5)	38.0–41.1
Birthweight (g)			3571	(527)	2680–4955
Placenta weight (g)^c^			656	(160)	341–1115
Birth/placental weight ratio			5.6	(1.0)	4.2–8.1
Sex (boys)	19	47.5			
Apgar 5 min	40		9.5	(0.7)	7–10

^a^One woman stopped smoking when pregnancy was confirmed in first trimester

^b^Defined after WHO-criteria. Plasma glucose ≥7.8mmol/L 2h after an oral glucose tolerance test of 75g glucose

^c^Untrimmed, without blood clots

Placenta extracted 6% of the glucose available per litre maternal blood passing, calculated by dividing the uteroplacental a-v glucose difference (0.29mmol/L) by the concentration in the radial artery (4.71mmol/L). By similar calculation the fetus consumed approximately 10% of glucose per litre blood flowing in the fetal-placental circulation.

**Fig 2:**
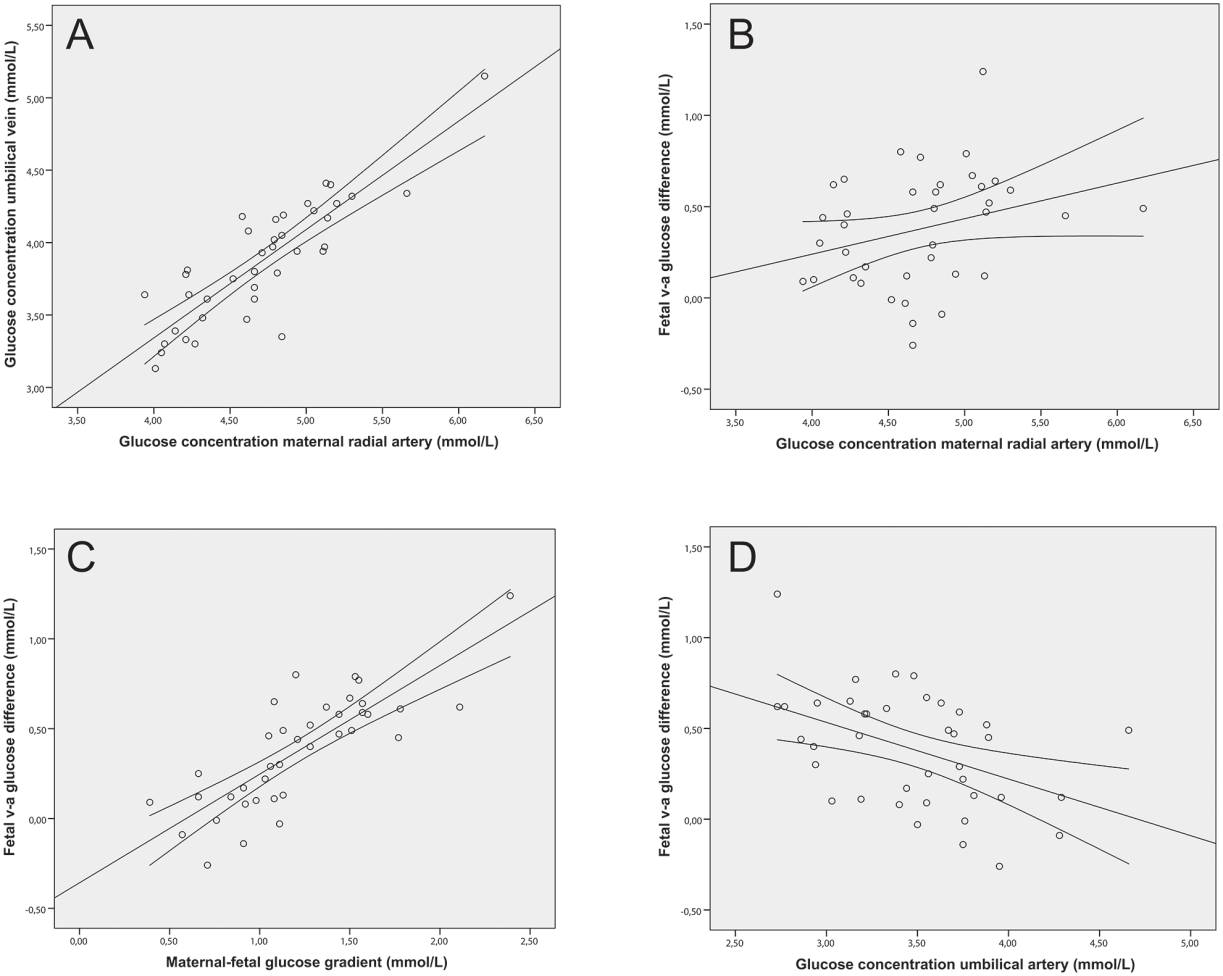
Correlations between maternal and fetal glucose concentrations and gradients. A & B. The maternal arterial glucose concentration was significantly correlated to the glucose concentration in the umbilical vein, r = 0.86, *p*<0.001, but not to the fetal v-a glucose difference, r = 0.3, *p* = 0.07. C & D. The fetal v-a glucose difference was significantly correlated to the maternal-fetal glucose gradient r = 0.8, *p*<0.001 and the glucose concentration in the umbilical artery r = −0.38, *p* = 0.017.

Maternal arterial and fetal venous concentrations of glucose were highly correlated (r = 0.86, *p*<0.001). Maternal arterial glucose concentration was not significantly correlated to the fetal v-a glucose difference (r = 0.3, *p* = 0.07). The uteroplacental a-v glucose difference was neither correlated to the level of glucose in the umbilical vein, nor the fetal v-a glucose difference. The maternal-fetal gradient was highly correlated with the fetal v-a difference (r = 0.8, *p*<0.001). The maternal-fetal glucose gradient was also correlated with the glucose concentration in the umbilical artery (r = -0.38, *p* = 0.017), whereas it was no correlation was found with the glucose concentration in the umbilical vein. Similarly, the fetal v-a glucose difference was only correlated with the glucose concentration in the umbilical artery (r = -0.45, *p* = 0.004), but not with the glucose concentration in the umbilical vein (Fig. 2 and S1 Table).

The glucose concentration in the umbilical vein was not related to birthweight or placental weight, whereas correlation between the fetal v-a glucose difference and birthweight reached significance, (r = 0.32, *p* = 0.049) (S1 Table).

Insulin concentrations are given in Table 2. We found no correlation between maternal and fetal insulin values (rho = −0.1, *p* = 0.57). Maternal and fetal concentrations of insulin were not statistically different. On the maternal side glucose and insulin concentrations were correlated (r = 0.52, *p* = 0.001). On the fetal side, however, no such correlation was found (r = 0.12, *p* = 0.48), but fetal insulin was correlated to the fetal v-a glucose difference (r = 0.43, *p* = 0.007). Fetal insulin was also correlated to placental weight (r = 0.46, *p* = 0.003) and birthweight (r = 0.58, *p*<0.001) (S1 Table).

**Table 2:** Glucose and insulin concentrations.

**Vessel**	**Mean glucose mmol/L**	**(SD)**	**Median insulin pmol/L**	**(IQR)**
**Radial artery**	4.71	(0.48)	56.0	(30.3–83.2)
**Antecubital vein**	4.56	(0.45)	49.6	(26.4–67.2)
**Uterine vein**	4.41	(0.45)	38.5	(23.9–55.7)
**Umbilical vein**	3.87	(0.41)	62.4	(36.9–92.0)
**Umbilical artery**	3.48	(0.45)	58.5	(34.8–85.9)

## Discussion

The 4-vessel sampling method was successfully implemented in our maternity ward and we obtained both arterial and venous samples from 40 mother-infant pairs.

### Glucose

We found the expected arterio-venous differences in glucose concentrations on both sides of the placenta, reflecting uteroplacental uptake of glucose on the maternal side and fetal uptake of glucose on the fetal side. The concentrations were higher on the maternal than on the fetal side, demonstrating a transplacental maternal-fetal gradient also in the fasting state.

The maternal-fetal gradient is recognized as the main driving force behind the facilitated diffusion of glucose over the placenta. Even though we found the expected strong correlation between maternal and umbilical glucose concentrations we found no significant association between maternal concentrations and fetal v-a difference. Moreover there was no correlation between the uteroplacental a-v difference and the fetal v-a difference. The maternal-fetal gradient and the fetal v-a difference were strongly correlated. Taken together, these findings suggest that fetal v-a glucose difference is not just affected by incoming glucose. This notion is supported by data from experimental studies which demonstrate that fetal glucose v-a difference is not simply related to the maternal concentration, but separately regulated by the fetal concentration of glucose [12]. The maternal-fetal gradient may be increased either by elevated glucose on the maternal side or by lower concentrations on the fetal arterial side [12, 13]. The latter concentration is largely determined by the fetal glucose consumption, demonstrating a way by which the fetus itself may regulate the transplacental glucose transport. The lack of correlation between the uteroplacental a-v difference and the fetal v-a difference might be due to the fact that a significant fraction of the glucose is metabolized by the placenta and the uterus [13, 18]. It has been demonstrated that placental glucose uptake and metabolism is related to fetal arterial glucose concentration [21]. Our results add to the complexity of nutrient transfer from the mother to the fetus. The assumption that controlling maternal blood glucose ensures control of fetal glucose uptake does not take into account the metabolism and consumption of nutrients that take place in the placenta. This is reflected in the clinical observation that even diabetic mothers with optimal blood glucose control have increased risk of delivering large for gestational age infants.

### Insulin

In this study glucose and insulin concentrations were not correlated in the fetal circulation, as opposed to in the maternal circulation. The physiological relation between insulin and glucose in the fetus is different from the mother. In the mother nutrients pass through the gut and the gastrointestinal hormones mediates additional stimulation of the pancreatic secretion of insulin in response to the meal. The fetus, on the other hand, has ongoing intravenous supply of glucose, thus bypassing the gut response [22]. The pancreatic response to a rise in blood glucose is different in fetal life. In experimental ovine studies Hay et al. concluded that basal insulin secretion has little effect on fetal glucose metabolism, but that glucose stimulated insulin secretion does occur, increases over gestation and is enhanced by pulsatile hyperglycaemia [12]. As our mothers were in a fasting state it is possible that the glucose stimulated insulin secretion played a limited role at the time of sampling.

### Comparison with other studies

Most previous studies of glucose and insulin concentrations in the human uteroplacental unit have been conducted during vaginal delivery, making comparison to our study difficult because of the non-fasting state and the physiological stress. These original studies have however demonstrated a consistent correlation between the maternal glucose concentration and glucose concentration in the umbilical vein, and also explored the relationship between fetal glucose and insulin [23, 24]. Several studies have demonstrated an inconsistent fetal insulin response to glucose. This has led to the notion that insulin is primarily a growth promoting factor in the fetus [10, 23, 25]. In our study fetal insulin was indeed positively correlated to both birthweight and placental weight. In experimental studies fetal insulin infusions led to reduced fetal glucose concentration, but increased fetal glucose v-a difference (mg min^-1^kg^-1^) [26]. Interestingly, fetal insulin was also associated with the fetal v-a glucose difference in our material.

We have found three other human studies of fetal v-a glucose difference comparable to our setting, which is fasting women delivered by elective caesarean section near term. Only one study comprising 8 women had complete data on glucose and insulin in arterial and venous vessels on both the maternal and the fetal side of the placenta [20]. One study found a fetal v-a glucose difference comparable to our study, but lacked data from the maternal arterial circulation [17]. Despite lower maternal arterial glucose levels than in our study, the two other studies reported larger fetal v-a difference, uteroplacental a-v difference and maternal fetal gradient [20, 27] (S1 Table). These two studies had relatively lower glucose concentration in the umbilical artery which contributed in creating the larger fetal v-a difference and maternal-fetal gradient. There is no consistent difference in insulin levels which can explain these differences. The birthweight was similar between the studies, but there was a difference in the placental efficiency measured as birth/placental weight ratio.

Measurements of concentrations and blood flow (ml/min) on both sides of the placenta makes it possible to quantify the actual mass (g) of nutrients delivered to, taken up by and transported over the uteroplacental unit, as well as the mass consumed by the fetus. Zamudio et al. included these flow measurements on the maternal and fetal side of the placenta [27]. They found a significant relation between fetal glucose in the umbilical vein (mmol min^-1^kg^-1^) and fetal glucose v-a difference (μmol min^-1^kg^-1^), both multiplied with umbilical blood flow and normalized to birthweight. They did not report if fetal v-a difference was correlated with uteroplacental glucose a-v difference at the maternal side of the placenta or if it was correlated with glucose concentrations in the umbilical artery.

### Strengths and limitations

The current study is limited by lack of maternal and fetal blood flow data, and thus compares glucose delivered per liter blood passing the placenta. In particular, flow measurements in the uteroplacental circulation is methodologically demanding and published data so far show a large divergence that may include considerable variations beyond the biological ones [28–30]. Furthermore, the flow assessment should be performed as close as possible in time to the blood sampling. This is logistically highly requiring in a busy hospital setting, but never the less necessary in future studies.

Our study included women scheduled for elective caesarean section and hence our sample has higher age and parity than the average pregnant population. The fasting state limits interpretation of the results. The cross-sectional design near term gives no opportunity to evaluate longitudinal changes or nutritional transport at earlier gestational ages.

The strength of this study is the acquisition of human in vivo blood samples from 40 individuals on both sides of the uteroplacental unit, under standardized conditions. No complications were observed neither when sampling from the radial artery nor from the uterine vein. The samples were handled by strict procedures.

## Conclusion

In conclusion, our method provides novel data on human placental glucose transfer in vivo and may be applied to any nutrients or substance of interest. We did not find support for the hypothesis that fetal v-a glucose difference, as a reflection of fetal consumption, is directly related to the supply of glucose from the maternal circulation. Our findings indicate that the transplacental transfer, measured as the maternal-fetal gradient, is significantly influenced by the fetal v-a glucose difference and not merely dependent on maternal glucose concentration or the glucose a-v difference on maternal side.

## Supporting Information

S1 Table Correlations between glucose concentrations and gradients, insulin, placental weight and birthweight. (DOC)

S2 Table Maternal and fetal glucose concentrations and gradients. Comparisons between four studies.
(DOCX)
