# Supplementary material for: Placental Glucose Transfer: A Human In Vivo Study
Source: PLoS One. 2015 Feb 13;10(2):e0117084. doi: 10.1371/journal.pone.0117084 (PMC4334523; doi:10.1371/journal.pone.0117084)
Supplement: S1 Table — (DOC) [file pone.0117084.s001.doc]

|  | **[G]_ma_** | **ΔG_ma-mv_** | **[G]_fv_** | **[G]_fa_** | **ΔG_fv-fa_** | **∆G_ma-fa_** | **[I]_ma_** | **[I]_fv_** | **Placental**  **weight (g)** | **Birth-**  **weight (g)** |
| --- | --- | --- | --- | --- | --- | --- | --- | --- | --- | --- |
| **[G]_ma_** | 1 |  |  |  |  |  |  |  |  |  |
| **ΔG_ma-mv_** | 0.35  *p*=0.03 | 1 |  |  |  |  |  |  |  |  |
| **[G]_fv_** | 0.86  *p*<0.001 | 0.16  *p*=0.33 | 1 |  |  |  |  |  |  |  |
| **[G]_fa_** | 0.60  *p*<0.001 | 0.03  *p*=0.88 | 0.74  *p*<0.001 | 1 |  |  |  |  |  |  |
| **ΔG_fv-fa_** | 0.30  *p*=0.07 | 0.18  *p*=0.28 | 0.27  *p*=0.10 | -0.45  *p*=0.004 | 1 |  |  |  |  |  |
| **∆G_ma-fa_** | 0.51  *p*=0.001 | 0.37  *p*=0.02 | 0.20  *p*=0.24 | -0.38  *p*=0.017 | 0.81  *p*<0.001 | 1 |  |  |  |  |
| **[I]_ma_** | 0.52  *p*=0.001 | 0.21  *p*=0.20 | 0.59  *p*<0.001 | 0.41  *p*=0.011 | 0.20  *p*=0.24 | 0.16  *p*=0.35 | 1 |  |  |  |
| **[I]_fv_** | 0.15  *p*=0.37 | 0.03  *p*=0.88 | 0.12  *p*=0.48 | -0.19  *p*=0.25 | 0.43  *p*=0.007 | 0.41  *p*=0.012 | -0.03*^a^*  *p*=0.87 | 1 |  |  |
| **Placental weight (g)** | -0.07  *p*=0.67 | 0.09  *p*=0.58 | -0.03  *p*=0.83 | -0.14  *p*=0.41 | 0.16  *p*=0.32 | 0.06  *p*=0.71 | 0.12  *p*=0.47 | 0.46  *p*=0.003 | 1 |  |
| **Birthweight (g)** | -0.01  *p*=0.96 | 0.03  *p*=0.87 | 0.03  *p*=0.86 | -0.19  *p*=0.26 | 0.32  *p*=0.049 | 0.22  *p*=0.19 | 0.26  *p*=0.12 | 0.58  *p*<0.001 | 0.81  *p*<0.001 | 1 |

Pearson’s correlation coefficient, unless otherwise stated

^a^Spearman’s correlation coefficient

[G] = concentration of glucose, [I] = concentration of insulin

ma = maternal arterial, _mv =_ maternal venous, _fv =_ fetal venous, _fa =_ fetal arterial

ΔG_ma-mv =_ maternal a-v glucose difference, ΔG_fv-fa =_ fetal v-a glucose difference, ∆G_ma-fa =_ maternal-fetal glucose gradient
