# Supplementary material for: Placental Glucose Transfer: A Human In Vivo Study
Source: PLoS One. 2015 Feb 13;10(2):e0117084. doi: 10.1371/journal.pone.0117084 (PMC4334523; doi:10.1371/journal.pone.0117084)
Supplement: S2 Table — Comparisons between four studies. (DOCX) [file pone.0117084.s002.docx]

|  | Maternal  concentrations | | | | Fetal concentrations | | Gradients | | |
| --- | --- | --- | --- | --- | --- | --- | --- | --- | --- |
|  | Radial artery | Arterialized  venous | Antecubital vein | Uterine vein | Umbilical vein | Umbilical artery | Utero-placental | Fetal | Maternal-fetal |
| Metzger^a^  et al. | 4.38 |  |  | 3.72 | 3.51 | 2.91 | 0.65 | 0.62 | 1.45 |
| Zamudio  et al. |  | 4.3 | 3.2 |  | 3.5 | 2.8 | 0.85^b^ | 0.74 | 1.51^c^ |
| Kuo |  |  | 4.43 |  | 4.15 | 3.81 |  | 0.33 |  |
| Holme et al  (current study) | 4.71 |  | 4.56 | 4.41 | 3.87 | 3.48 | 0.29 | 0.38 | 1.22 |

^a^ Including one twin pregnancy

^b^ Gradient between arterialized venous and antecubital vein glucose concentrations.

^c^ Gradient between maternal arterialized venous and umbilical arterial glucose concentrations
